# The G2 checkpoint inhibitor CBP-93872 increases the sensitivity of colorectal and pancreatic cancer cells to chemotherapy

**DOI:** 10.1371/journal.pone.0178221

**Published:** 2017-05-30

**Authors:** Tsutomu Iwata, Tairin Uchino, Ayako Koyama, Yoshikazu Johmura, Kenichi Koyama, Takuya Saito, Seiji Ishiguro, Takashi Arikawa, Shunichiro Komatsu, Masahiko Miyachi, Tsuyoshi Sano, Makoto Nakanishi, Midori Shimada

**Affiliations:** 1 Department of Cell Biology, Graduate School of Medical Sciences, Nagoya City University, Nagoya, Aichi, Japan; 2 Department of Gastroenterological Surgery, Aichi Medical University School of Medicine, Nagakute, Japan; 3 Division of Cancer Cell Biology, Department of Cancer Biology, Institute of Medical Science, The University of Tokyo, Minato-ku, Tokyo, Japan; University of South Alabama Mitchell Cancer Institute, UNITED STATES

## Abstract

CBP-93872 suppresses maintenance of DNA double-stranded break-induced G2 checkpoint, by inhibiting the pathway between ataxia-telangiectasia mutated (ATM) and ATM- and Rad3-related (ATR) activation. To examine the potential use of CBP-93872 for clinical applications, we analyzed the synergistic effects of platinum-containing drugs, oxaliplatin and cisplatin, pyrimidine antimetabolites, gemcitabine and 5-fluorouracil (5-FU), in combination with CBP-93872, on cell lethality in colorectal and pancreatic cancer cell lines. Treatment with CBP-93872 significantly increased cancer cell sensitivities to various chemotherapeutic agents tested through suppression of checkpoint activation. Our results thus reveal that combination treatment of CBP-93872 with known chemotherapeutic agents inhibits phosphorylation of ATR and Chk1, and induces cell death.

## Introduction

All mammalian cells are continuously exposed to endogenous and exogenous DNA damaging stresses, such as ultraviolet (UV) rays, oxidative stress and ionizing radiation (IR). To maintain genomic stability against these stresses, cells activate a global signaling network, termed DNA damage response (DDR); which in turn leads to cell cycle arrest, apoptosis, and premature senescence [1]. Upon DNA damage, abnormal DNA structures are rapidly sensed, and DNA damage signals are transmitted to downstream effectors via the phosphatidylinositol 3-kinase-related protein kinases (PIKKs) ATM (ataxia telangiectasia mutated) and ATR (ATM and Rad3 related). These kinases phosphorylate multiple key regulators to mediate various cellular responses [2].

One such critical downstream regulator is Chk1 (checkpoint kinase 1). Following DNA damage and stalled DNA replication, Chk1 is phosphorylated at S317 and S345, mainly by ATR. Furthermore, subcellular localization of Chk1 is altered upon phosphorylation, allowing Chk1-mediated phosphorylation of important cell cycle modulators including p53 and Cdc25 phosphatases. This triggers multiple downstream events such as cell cycle arrest, and transcriptional repression [3–5]. Chk1 is thus essential for the S-phase, and G2, DNA damage checkpoints [6–8]; and also DNA replication checkpoints [9, 10].

Transient cell cycle arrest after DNA damage is mediated by two distinct signaling pathways; one is the p53-p21-dependent G1 checkpoint [11], and the other is the Chk1-Cdc25-dependent G2 checkpoint [12, 13]. Given that most cancer cells lack functional p53, and are thus defective in the G1 checkpoint, effective DNA repair of these cancer cells and their survival depend on the G2 checkpoint. G2 checkpoint inhibitors, therefore, might be used as chemosensitizers of known anticancer therapies for p53-deficient cancer cells [14–16].

Indeed, platinum-based chemotherapy is now widely used for treatment of various cancers [17]. Colon and pancreatic cancers are leading causes of cancer-related death worldwide. Chemotherapeutic agents such as oxaliplatin and gemcitabine are currently used for colon or pancreatic cancer treatments, respectively. It is, however, widely known that cancer cells eventually acquire chemoresistance against these drugs [18–20]. To overcome such resistances, combinatorial therapy- using two or more chemotherapeutic agents together, has become a common strategy; to optimize efficacy of cancer treatment, and also reduce toxicity toward normal cells.

Combinatorial therapy of platinum-based drugs with other chemicals are now being commonly employed for treatment of various types of cancers [21]. One such chemical is FOLFIRINOX (folinic acid, 5-fluorouracil, irinotecan, and oxaliplatin), which improves overall survival in metastatic pancreatic cancer [22]. Indeed, beneficial roles of FOLFIRINOX treatment in combination with bevacizumab, has been reported in metastatic colorectal cancers [23]. Similarly, administration of platinum-drugs in combination with Nivolumab, also improved survival in advanced Non-Small-Cell Lung Cancers [24]. Despite such improvements, however, it is also clear that development of more effective therapeutic strategies is required to enhance clinical efficacy of existing chemotherapeutic agents.

Using p53-deficient cell based screening, we previously identified CBP-93872 as a promising G2 checkpoint inhibitor [25]. CBP-93872 specifically suppresses the maintenance, but not initiation, of DNA double strand break (DSB)-induced G2 checkpoint; by inhibiting Nbs1-dependent activation of ATR [26]. To evaluate the potential use of this drug for clinical application, we explored the synergistic effects of various anticancer agents in combination with CBP-93872, on cell lethality in p53-deficient colorectal cancer (HT29), and pancreatic cancer cells (Panc-1).

## Results

### Combined treatment of CBP-93872 with oxaliplatin, cisplatin, 5-FU or gemcitabine effectively suppresses cell growth

**Fig 1:**
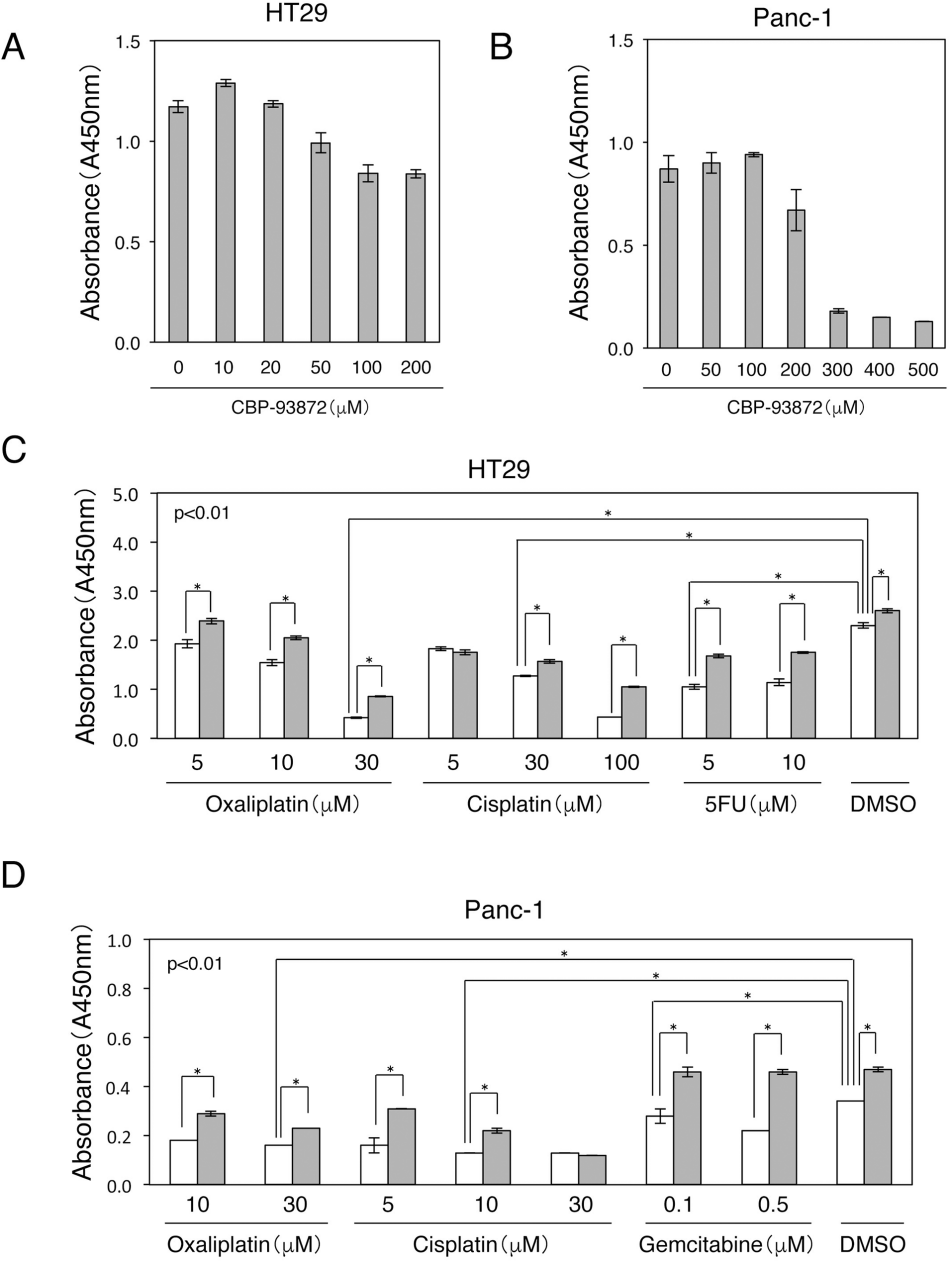
Combined treatments of CBP-93872 with oxaliplatin, cisplatin, 5-FU, or gemcitabine effectively suppresses cell growth. (A) HT29 cells were treated with the indicated concentrations of CBP-93872 for 72 hrs, followed by WST-1 assay. Data are presented as means ± SD (n = 3). (B) Panc-1 cells were treated with the indicated concentrations of CBP-93872 for 72 hrs, followed by WST-1 assay. Data are presented as means ± SD (n = 3). (C) HT29 cells were treated with CBP-93872 (50 μM) in combination with indicated concentrations of oxaliplatin, cisplatin or 5-FU for 72 hrs, followed by WST-1 assay. (D) Panc-1 cells were treated with CBP-93872 (200 μM) in combination with indicated concentrations of oxaliplatin, cisplatin or gemcitabine for 72 hrs, followed by WST-1 assay. The black bars show individual treatments; while the white bars show combined treatment with CBP-93872. Data are presented as means ± SD (n = 3). Statistical significance was calculated using Student’s *t-*test (*, p < 0.01) (C, D).

To examine the synergistic effects of CBP-93872 with various chemotherapeutic agents on cell death, we first determined the minimum concentrations of CBP-93872 to suppress HT29 or Panc-1 cell proliferation, using the WST-1 assay. We found that CBP-93872 suppressed cell proliferation, at concentrations greater than 50 μM (HT29) or 200 μM (Panc-1), 72 hrs after the treatment (Fig 1A and 1B).

Oxaliplatin and cisplatin are commonly used for treating colorectal and pancreatic cancers. Both of these drugs are platinum-containing compounds that produce bulky DNA adducts and DNA cross-links. Repair of such crosslinks frequently results in the generation of DSBs. In contrast, 5-FU induces replication fork arrest, leading to an S-phase block, and is widely used as an antimetabolite for colorectal cancer. To investigate the combined effect of CBP-93872 and various anticancer drugs on cell proliferation, we treated HT29 cells with oxaliplatin (5–30 μM), cisplatin (5–100 μM) or 5-FU (5–10 μM), together with CBP-93872 (50 μM). Indeed, combined treatment of CBP-93872 together with oxaliplatin, cisplatin or 5-FU significantly reduced HT29 cells proliferation in almost all concentrations; being more effective at high concentrations (Fig 1C).

Gemcitabine is an analog of deoxycytidine that inhibits DNA synthesis. This is also the major drug used for clinical intervention of pancreatic cancers. To investigate the role of gemcitabine to suppress the growth of a pancreatic cancer cell line, we treated Panc-1 cells with oxaliplatin (10–30 μM), cisplatin (5–30 μM) or gemcitabine (0.1–0.5 μM), in combination with CBP-93872 (200 μM). We again observed that combined treatment of CBP-93872 with oxaliplatin, cisplatin or gemcitabine efficiently inhibited cell proliferation (Fig 1D). Taken together, these results indicate that CBP-93872 acts as a chemosensitizer with platinum-containing drugs or pyrimidine antimetabolites.

### CBP-93872 enhances oxaliplatin, cisplatin, gemcitabine or 5-FU mediated apoptosis

**Fig 2:**
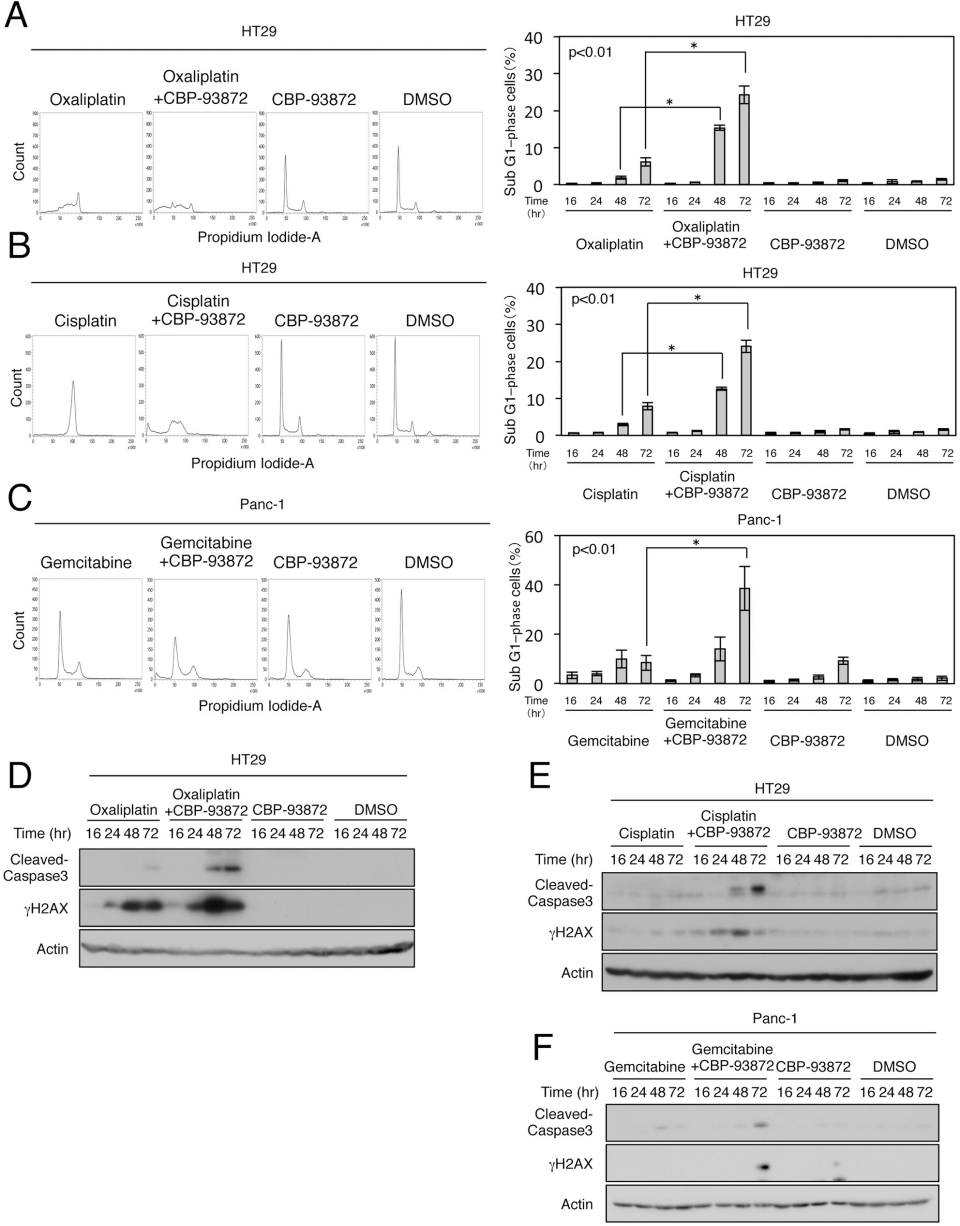
CBP-93872 enhances oxaliplatin-, cisplatin- and gemcitabine-induced apoptosis in HT29 cells or Panc-1 cells. (A, B) HT29 cells were treated with oxaliplatin (30 μM) (A) or cisplatin (30 μM) (B) in the presence or absence of CBP-93872 (50 μM). Cells were harvested at 72 hrs, fixed and subjected to FACS analysis (left panels). The percentages of cells in sub-G1 phase are shown in the panels on the right. Data are presented as means ± SD (n = 3). Statistical significance was calculated using Student’s *t-*test (*, p < 0.01). (C) Panc-1 cells were treated with gemcitabine (0.1 μM) in the presence or absence of CBP-93872 (200 μM). (D-F) HT29 cells or Panc-1 cells, were collected at the times indicated. Total cell extracts were subjected to immunoblotting, using the indicated antibodies.

We next examined whether suppression of cell proliferation by combined treatment of CBP-93872 with oxaliplatin, cisplatin, gemcitabine or 5-FU was mediated via apoptosis. Indeed, HT29 cells treated with both CBP-93872 and oxaliplatin showed significant increase in sub-G1 cell population (from 6.1% to 24.3%) (Fig 2A). Administration of CBP-93872 and cisplatin, or gemcitabine also produced similar effects in HT29 cells or Panc-1 cells (Fig 2B and 2C). Importantly, combined treatments with CBP-93872 markedly increased cisplatin-induced apoptosis (from 7.9% to 24.0%), in HT29 cells. CBP-93872 similarly increased gemcitabine-induced apoptosis (from 8.4% to 38.5%) in Panc-1 cells, and 5-FU-induced apoptosis in HT29 cells (from 5.8% to 19.5%) (S1 Fig). These results indicate that CBP-93872 sensitizes oxaliplatin, cisplatin, gemcitabine or 5-FU-induced apoptosis in cancer cell lines.

Consistent with the above observations, cleaved caspase 3- a marker of apoptosis, was abundantly detected after combined treatment with CBP-93872 (Fig 2D–2F, S1 Fig). In addition, the level of γH2AX- a marker of DNA DSBs, was also elevated after combined treatment with CBP-93872 (Fig 2D–2F).

### CBP-93872 abrogates oxaliplatin or cisplatin induced G2 checkpoint

**Fig 3:**
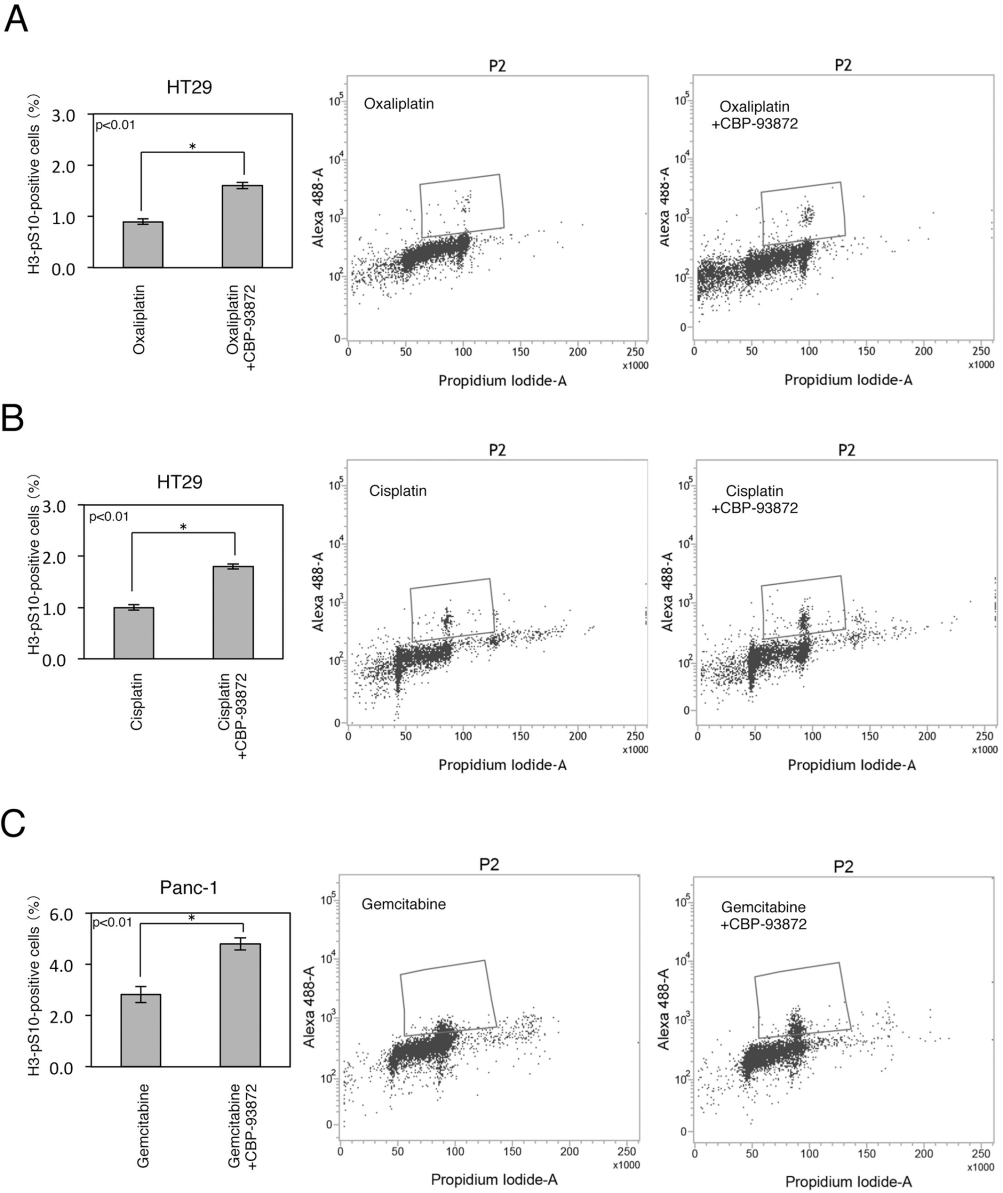
CBP-93872 inhibits maintenance of G2 and S-phase checkpoints. (A, B) HT29 cells were treated with oxaliplatin (30 μM) (A), or cisplatin (30 μM) (B) in the presence or absence of CBP-93872 (50 μM). Nocodazole (500 nM) was added to inhibit the exit of cells from mitosis. Cells were harvested at 48 hrs, fixed, and stained with anti-H3-pS10 antibodies to determine the mitotic index. Mitotic indices (left) and typical examples (right) are shown. Data are presented as means ± SD (n = 3). Statistical significance was calculated using Student’s *t-*test (*, p < 0.01). ((C) Panc-1 cells were treated with gemcitabine (0.1 μM) in the presence or absence of CBP-93872 (200 μM) with nocodazole (500 nM).

We previously reported that CBP-93872 specifically suppresses IR-induced G2 checkpoint [26]. We asked whether this was also the case for oxaliplatin or cisplatin. Indeed, we observed a higher mitotic index after CBP-93872 treatment in combination with oxaliplatin or cisplatin, in HT29 cells (Fig 3A and 3B). These findings thus indicated that CBP-93872 inhibits oxaliplatin or cisplatin induced G2 checkpoint.

It has also been reported that gemcitabine induces S-phase arrest to prevent premature mitosis [27]. Thus, gemcitabine-treated cells showed a decreased mitotic index. This reduction was significantly attenuated by a concomitant use of CBP-93872 in Panc-1 cells (Fig 3C), indicating that the S-phase checkpoint triggered by gemcitabine was also abolished by CBP-93872.

### CBP-93872 reduces the levels of phosphorylated ATR and Chk1

**Fig 4:**
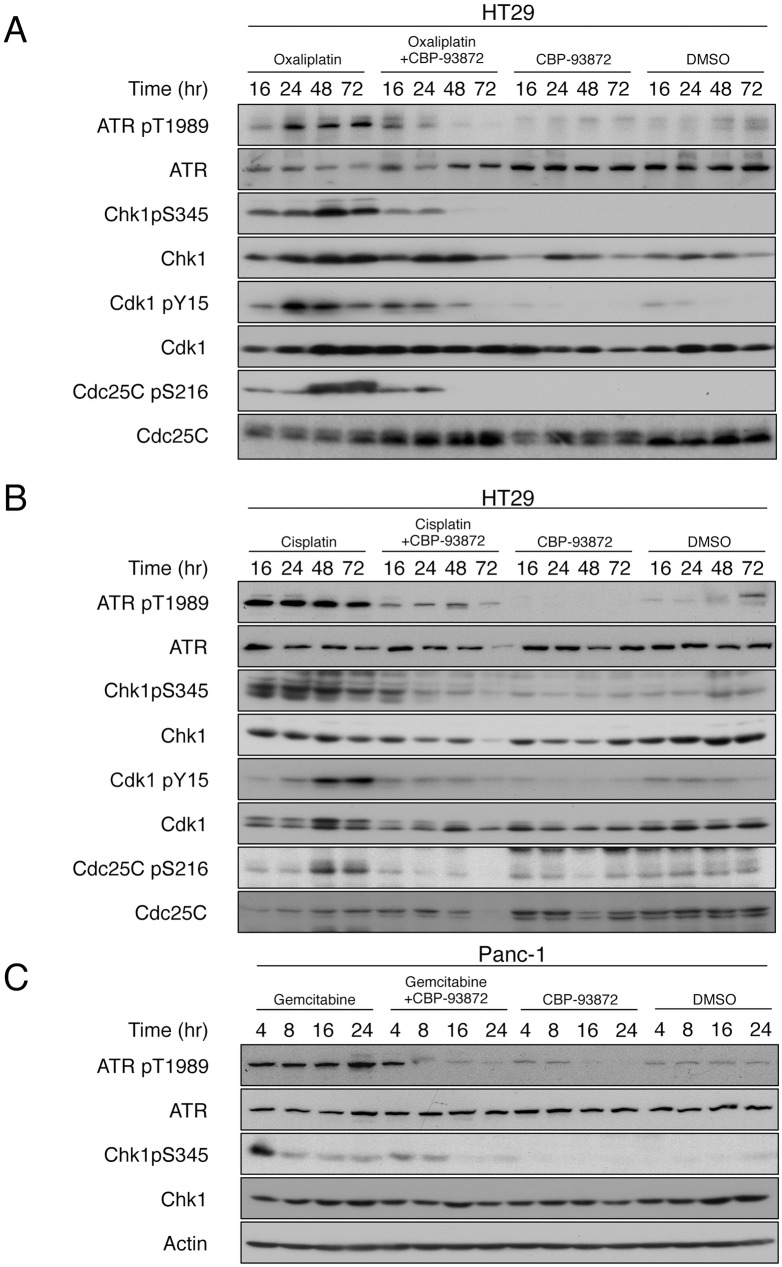
CBP-93872 reduces the levels of oxaliplatin-, cisplatin- and gemcitabine-induced phosphorylations of ATR and Chk1. (A, B) HT29 cells were treated with oxaliplatin (30 μM) (A), or cisplatin (30 μM) (B) in the presence of absence of CBP-93872 (50 μM). Cells were harvested at the times indicated, and total cell extracts were subjected to immmunoblotting using indicated antibodies. (C) Panc-1 cells were treated with gemcitabine (0.1 μM), in the presence or absence of CBP-93872 (200 μM).

In order to elucidate the molecular basis underlying increased level of apoptosis by combined treatment of CBP-93872 with other anticancer drugs, we examined whether CBP-93872 treatment affected ATR activation or Chk1 phosphorylation. ATR activation, measured by auto-phosphorylation at T1989, was readily detected in cells treated with oxaliplatin or cisplatin alone (25) (Fig 4A and 4B and S2 Fig). Furthermore, phosphorylation of Chk1 at S345 was also detected under the same conditions (Fig 4A and 4B and S2 Fig).

As expected, combined treatment with CBP-93872 strongly inhibited ATR activation, and Chk1 phosphorylation, induced by oxaliplatin or cisplatin alone. The G2 checkpoint is mediated at least in part by Cdc25C phosphorylation at S216, and the consequent inhibition of Cdk1 by its phosphorylation at Y15/Y14. Consistent with these observations, combined treatment of CBP-93872, with anticancer drugs, reduced the levels of phosphorylation of Cdk1 and Cdc25C (Figs 4A and 5B). We also found that CBP-93872 reduced the levels of phosphorylation of ATR and Chk1, in gemcitabine-treated Panc-1, or 5-FU-treated HT29 cells (Fig 4C, S2 Fig).

## Discussion

Increased chemo- or radio-resistance, or both, causes major difficulties in the treatment and management of malignant cancers. It is believed that cancer cells gain therapeutic resistances via activation, and enhancement, of specific DNA repair pathways [28]. Administration of cytotoxic agents, in combination with chemosensitizers: such as inhibitors of cell cycle checkpoints or DNA repair pathways, is therefore of great importance for development of effective cancer therapies. These combined therapies likely result in synthetic lethality, in specific types of cancer cells.

In addition, several checkpoint kinase inhibitors have been extensively studied, and have entered clinical trials [29]. For example, specific ATR inhibitors (VE-821 and NU6027) were shown to enhance cytotoxicity of cancer cells in combination with multiple DNA damaging agents. These inhibitors are currently being evaluated in Phase 1 clinical developments [30]. Furthermore, inhibitors of DNA damage mediators PARP1 (Poly [ADP-ribose] polymerase 1) and WEE1 (WEE1 G2 Checkpoint Kinase) have also been developed, and tested via clinical studies [31].

Most of the molecular targets of these inhibitors, however, are also important for the survival of normal cells. As a result, these inhibitors could cause unfavorable toxicity in normal cells. Consistent with this notion, UCN-01: which inhibits Chk1, is not suitable for clinical interventions owing to unpredictable toxicity results obtained in Phase 1 trials [32].

We have previously identified that CBP-93872 inhibits ssDNA-induced ATR activation, by activating multiple substrates that regulate DNA repair and cell cycle arrest [26]. It has also been reported that Chk1 modulates DNA repair via regulation of the homologous recombination repair protein Rad51 [33]. In addition, replication stress caused by chemotherapeutic agents, such as oxaliplatin, cisplatin and gemcitabine, activates ATR. ATR in turn phosphorylates multiple downstream substrates that orchestrate DNA damage responses. Cells may survive replication stress by preventing firing of replication origins, stabilizing stalled replication forks, and promoting DNA repair and cell cycle checkpoints. Such processes are likely disrupted by concomitant treatments of CBP-93872 with chemotherapeutic agents used in this study. Thus, cell death induced by CBP-93872 could be mediated through destabilization of one or more pathways mentioned above.

Anticancer drugs such as cisplatin, oxaliplatin, 5-FU and gemcitabine are commonly used to treat digestive tract cancer. Indeed, combined use of these drugs have been extensively employed for clinical interventions [34–37]. While response rates and progression-free survival has improved by these therapies, no ideal combination of agents with fewer side effects and broader cytotoxicity has yet emerged.

CBP-93872 specifically suppresses G2 checkpoint through inhibition of DSB-dependent ATR activation [25, 26], and therefore is expected to enhance the effect of anticancer drugs in p53-deficient cancer cells, as the G2 checkpoint is required for survival in these cells. Importantly, CBP-93872 should have little impact on normal cells, as these cells possess a functional p53-p21 pathway. Our study indicates that CBP-93872 might be a potential candidate as a chemosensitizer, for combined use with existing anticancer agents such as oxaliplatin, cisplatin, gemcitabine, or 5-FU.

## Materials and methods

### Cell culture

The HT29 human colorectal cancer cell line was cultured in McCoy’s 5A medium (Thermo Fisher Scientific), supplemented with 10% fetal bovine serum (FBS) and 1% penicillin-streptomycin (PS). Panc-1 human pancreatic cancer cells were obtained from RIKEN BRC through the National Bio Resource Project of the MEXT, Japan. Panc-1 was grown in RPMI-1640 (Sigma-Aldrich) supplemented with 10% FBS and 1% PS. All cells were cultured at 37°C in 5% CO_2_.

### Reagents

CBP-93872 (Chugai Pharmaceutical company) was used at a final concentration of 50 μM (HT29) and 200 μM (Panc-1), unless otherwise indicated. Cisplatin (Wako) was used in a final concentration of 30 μM (HT29) and 10 μM (Panc-1), while the final concentrations of oxaliplatin (Wako), 5-FU (Wako), gemcitabine (Tokyo Chemical Industry), nocodazole (Sigma-Aldrich) were 30 μM, 5 μM, 0.1 μM, and 500 nM, respectively. DMSO (Sigma-Aldrich) was used as a control.

### WST-1 cell proliferation assay

The viability of the cells was determined using the Premix WST-1 cell proliferation assay (Roche Applied Science). Cell lines were seeded in 96-well plates (4.0×10^3^ cells/well), in 100 μL medium, and allowed to attach overnight. Following cellular adhesion, treatment with cisplatin, oxaliplatin, 5-FU, gemcitabine was performed. After 72 hrs, 10 μL WST-1 reagent was added to the plates followed by additional incubation for 1 hr. The absorbance reading of each well was measured using a microplate reader (iMARK; Bio-Rad), at a wavelength of 450 nm.

### Western blotting

Collected cells were suspended in SDS sample buffer, boiled for 5 min, separated by SDS-PAGE and transferred to a polyvinylidene difluoride membrane. Membranes were incubated overnight with primary antibodies, followed by 1 h incubation with secondary antibodies. The antibodies used for western blotting were ATR (sc-1887; Santa Cruz Biotechnology), phospho-ATR Thr1989 (128145; GeneTex, Inc.), Chk1 (C9358; Sigma-Aldrich), phospho-Chk1 Ser345 (2348; Cell Signaling Technology), Cdk1 (sc-54; Santa Cruz Biotechnology), phospho-Cdk1 Tyr15 (9111; Cell Signaling Technology), Cdc25C (sc-13138; Santa Cruz Biotechnology), phospho-Cdc25C Ser216 (9528; Cell Signaling Technology), Cleaved-caspase3 (9661; Cell Signaling Technology), γH2AX (61796; GeneTex, Inc.) and β-actin (ab6276; Abcam).

### Cell cycle analysis

Cells were harvested at 16, 24, 48, 72 hr after treatment, and fixed with 70% ethanol. Cell pellets were washed once with PBS, and stained with phospho-histone H3 Ser10 antibodies (06–570; Millipore) for 2 hr, followed by 30 min incubation with Alexa Fluor 488 secondary antibodies (Thermo Fisher Scientific), and counterstained with 0.1 mg/mL propidium iodide containing RNase for 30 min at 37°C. Cell cycle analysis was performed by flow cytometry using a BD FACSVerse^TM^ flow cytometer (BD Biosciences).

## Supporting information

S1 Fig Combined treatment of CBP-93872 with oxaliplatin, cisplatin, 5-FU, or gemcitabine effectively suppresses cell growth in HT29 or Panc-1 cells. (A) HT29 cells were treated with 5-FU (5 μM), in the presence or absence of CBP-93872 (50 μM). Cells were harvested at the times indicated, fixed and subjected to FACS analysis to determine the proportion of cells in sub-G1 phase. Data are presented as means ± SD (n = 3). Statistical significance was calculated using Student’s *t-*test (*, p < 0.01).
(B, C) Panc-1 cells were treated for the time indicated with oxaliplatin (30 μM) (B), or cisplatin (10 μM) (C), in the presence or absence of CBP-93872 (200 μM). Total cell extracts were analyzed by immunoblotting using the antibodies indicated.
(D) HT29 cells were treated and analyzed as in (A).
(TIF)

S2 Fig CBP-93872 reduces the levels of phosphorylation of ATR and Chk1 in HT29 and Panc-1 cells. (A) (B) Cells were treated as in S1 Fig, and total cell extracts were subjected to immmunoblotting using indicated antibodies.
(C) Experiments were performed as described in S1 Fig, and total cells extracts were subjected to immmunoblotting using indicated antibodies.
(TIF)

## References

[1] Jackson SP, Bartek J. The DNA-damage response in human biology and disease. Nature. 2009;461(7267):1071–8. PubMed Central PMCID: PMCPMC2906700. doi: 10.1038/nature08467 19847258 10.1038/nature08467 PMC2906700

[2] Shimada M, Nakanishi M. DNA damage checkpoints and cancer. J Mol Histol. 2006;37(5–7):253–60. doi: 10.1007/s10735-006-9039-4 16841236 10.1007/s10735-006-9039-4

[3] Niida H, Katsuno Y, Banerjee B, Hande MP, Nakanishi M. Specific role of Chk1 phosphorylations in cell survival and checkpoint activation. Mol Cell Biol. 2007;27(7):2572–81. PubMed Central PMCID: PMCPMC1899884. doi: 10.1128/MCB.01611-06 17242188 10.1128/MCB.01611-06 PMC1899884

[4] Shimada M, Niida H, Zineldeen DH, Tagami H, Tanaka M, Saito H, et al Chk1 is a histone H3 threonine 11 kinase that regulates DNA damage-induced transcriptional repression. Cell. 2008;132(2):221–32. doi: 10.1016/j.cell.2007.12.013 18243098 10.1016/j.cell.2007.12.013

[5] Shimada M, Nakanishi M. Checkpoints meet the transcription at a novel histone milestone (H3-T11). Cell Cycle. 2008;7(11):1555–9. doi: 10.4161/cc.7.11.6062 18469531 10.4161/cc.7.11.6062

[6] Mailand N, Falck J, Lukas C, Syljuasen RG, Welcker M, Bartek J, et al Rapid destruction of human Cdc25A in response to DNA damage. Science. 2000;288(5470):1425–9. 10827953 10.1126/science.288.5470.1425

[7] Liu Q, Guntuku S, Cui XS, Matsuoka S, Cortez D, Tamai K, et al Chk1 is an essential kinase that is regulated by Atr and required for the G(2)/M DNA damage checkpoint. Genes Dev. 2000;14(12):1448–59. PubMed Central PMCID: PMCPMC316686. 10859164 PMC316686

[8] Zachos G, Rainey MD, Gillespie DA. Chk1-deficient tumour cells are viable but exhibit multiple checkpoint and survival defects. EMBO J. 2003;22(3):713–23. PubMed Central PMCID: PMCPMC140744. doi: 10.1093/emboj/cdg060 12554671 10.1093/emboj/cdg060 PMC140744

[9] Guo Z, Kumagai A, Wang SX, Dunphy WG. Requirement for Atr in phosphorylation of Chk1 and cell cycle regulation in response to DNA replication blocks and UV-damaged DNA in Xenopus egg extracts. Genes Dev. 2000;14(21):2745–56. PubMed Central PMCID: PMCPMC317027. 11069891 10.1101/gad.842500 PMC317027

[10] Feijoo C, Hall-Jackson C, Wu R, Jenkins D, Leitch J, Gilbert DM, et al Activation of mammalian Chk1 during DNA replication arrest: a role for Chk1 in the intra-S phase checkpoint monitoring replication origin firing. J Cell Biol. 2001;154(5):913–23. PubMed Central PMCID: PMCPMC1255922. doi: 10.1083/jcb.200104099 11535615 10.1083/jcb.200104099 PMC1255922

[11] Mirzayans R, Andrais B, Scott A, Murray D. New insights into p53 signaling and cancer cell response to DNA damage: implications for cancer therapy. J Biomed Biotechnol. 2012;2012:170325 PubMed Central PMCID: PMCPMC3403320. doi: 10.1155/2012/170325 22911014 10.1155/2012/170325 PMC3403320

[12] Zhao H, Piwnica-Worms H. ATR-mediated checkpoint pathways regulate phosphorylation and activation of human Chk1. Mol Cell Biol. 2001;21(13):4129–39. PubMed Central PMCID: PMCPMC87074. doi: 10.1128/MCB.21.13.4129-4139.2001 11390642 10.1128/MCB.21.13.4129-4139.2001 PMC87074

[13] Zhao H, Watkins JL, Piwnica-Worms H. Disruption of the checkpoint kinase 1/cell division cycle 25A pathway abrogates ionizing radiation-induced S and G2 checkpoints. Proc Natl Acad Sci U S A. 2002;99(23):14795–800. PubMed Central PMCID: PMCPMC137498. doi: 10.1073/pnas.182557299 12399544 10.1073/pnas.182557299 PMC137498

[14] Fan S, Smith ML, Rivet DJ 2nd, Duba D, Zhan Q, Kohn KW, et al Disruption of p53 function sensitizes breast cancer MCF-7 cells to cisplatin and pentoxifylline. Cancer Res. 1995;55(8):1649–54. 7712469

[15] Goto H, Izawa I, Li P, Inagaki M. Novel regulation of checkpoint kinase 1: Is checkpoint kinase 1 a good candidate for anti-cancer therapy? Cancer Sci. 2012;103(7):1195–200. doi: 10.1111/j.1349-7006.2012.02280.x 22435685 10.1111/j.1349-7006.2012.02280.x PMC7659239

[16] Ma CX, Janetka JW, Piwnica-Worms H. Death by releasing the breaks: CHK1 inhibitors as cancer therapeutics. Trends Mol Med. 2011;17(2):88–96. doi: 10.1016/j.molmed.2010.10.009 21087899 10.1016/j.molmed.2010.10.009 PMC6905465

[17] Kelland L. The resurgence of platinum-based cancer chemotherapy. Nat Rev Cancer. 2007;7(8):573–84. doi: 10.1038/nrc2167 17625587 10.1038/nrc2167

[18] Doi T, Boku N, Kato K, Komatsu Y, Yamaguchi K, Muro K, et al Phase I/II study of capecitabine plus oxaliplatin (XELOX) plus bevacizumab as first-line therapy in Japanese patients with metastatic colorectal cancer. Jpn J Clin Oncol. 2010;40(10):913–20. PubMed Central PMCID: PMCPMC2947844. doi: 10.1093/jjco/hyq069 20462981 10.1093/jjco/hyq069 PMC2947844

[19] Yamada Y, Takahari D, Matsumoto H, Baba H, Nakamura M, Yoshida K, et al Leucovorin, fluorouracil, and oxaliplatin plus bevacizumab versus S-1 and oxaliplatin plus bevacizumab in patients with metastatic colorectal cancer (SOFT): an open-label, non-inferiority, randomised phase 3 trial. Lancet Oncol. 2013;14(13):1278–86. doi: 10.1016/S1470-2045(13)70490-X 24225157 10.1016/S1470-2045(13)70490-X

[20] Oettle H, Post S, Neuhaus P, Gellert K, Langrehr J, Ridwelski K, et al Adjuvant chemotherapy with gemcitabine vs observation in patients undergoing curative-intent resection of pancreatic cancer: a randomized controlled trial. JAMA. 2007;297(3):267–77. doi: 10.1001/jama.297.3.267 17227978 10.1001/jama.297.3.267

[21] Dasari S, Tchounwou PB. Cisplatin in cancer therapy: molecular mechanisms of action. Eur J Pharmacol. 2014;740:364–78. PubMed Central PMCID: PMCPMC4146684. doi: 10.1016/j.ejphar.2014.07.025 25058905 10.1016/j.ejphar.2014.07.025 PMC4146684

[22] Conroy T, Desseigne F, Ychou M, Bouche O, Guimbaud R, Becouarn Y, et al FOLFIRINOX versus gemcitabine for metastatic pancreatic cancer. N Engl J Med. 2011;364(19):1817–25. doi: 10.1056/NEJMoa1011923 21561347 10.1056/NEJMoa1011923

[23] Loupakis F, Cremolini C, Masi G, Lonardi S, Zagonel V, Salvatore L, et al Initial therapy with FOLFOXIRI and bevacizumab for metastatic colorectal cancer. N Engl J Med. 2014;371(17):1609–18. doi: 10.1056/NEJMoa1403108 25337750 10.1056/NEJMoa1403108

[24] Rizvi NA, Hellmann MD, Brahmer JR, Juergens RA, Borghaei H, Gettinger S, et al Nivolumab in Combination With Platinum-Based Doublet Chemotherapy for First-Line Treatment of Advanced Non-Small-Cell Lung Cancer. J Clin Oncol. 2016;34(25):2969–79. doi: 10.1200/JCO.2016.66.9861 27354481 10.1200/JCO.2016.66.9861 PMC5569693

[25] Harada N, Watanabe Y, Yoshimura Y, Sakumoto H, Makishima F, Tsuchiya M, et al Identification of a checkpoint modulator with synthetic lethality to p53 mutants. Anticancer Drugs. 2011;22(10):986–94. doi: 10.1097/CAD.0b013e328349dd43 21822123 10.1097/CAD.0b013e328349dd43

[26] Hirokawa T, Shiotani B, Shimada M, Murata K, Johmura Y, Haruta M, et al CBP-93872 inhibits NBS1-mediated ATR activation, abrogating maintenance of the DNA double-strand break-specific G2 checkpoint. Cancer Res. 2014;74(14):3880–9. doi: 10.1158/0008-5472.CAN-13-3604 24876101 10.1158/0008-5472.CAN-13-3604

[27] Morgan MA, Parsels LA, Parsels JD, Mesiwala AK, Maybaum J, Lawrence TS. Role of checkpoint kinase 1 in preventing premature mitosis in response to gemcitabine. Cancer Res. 2005;65(15):6835–42. doi: 10.1158/0008-5472.CAN-04-2246 16061666 10.1158/0008-5472.CAN-04-2246

[28] Bouwman P, Jonkers J. The effects of deregulated DNA damage signalling on cancer chemotherapy response and resistance. Nat Rev Cancer. 2012;12(9):587–98. doi: 10.1038/nrc3342 22918414 10.1038/nrc3342

[29] Visconti R, Della Monica R, Grieco D. Cell cycle checkpoint in cancer: a therapeutically targetable double-edged sword. J Exp Clin Cancer Res. 2016;35(1):153 PubMed Central PMCID: PMCPMC5037895. doi: 10.1186/s13046-016-0433-9 27670139 10.1186/s13046-016-0433-9 PMC5037895

[30] Weber AM, Ryan AJ. ATM and ATR as therapeutic targets in cancer. Pharmacol Ther. 2015;149:124–38. doi: 10.1016/j.pharmthera.2014.12.001 25512053 10.1016/j.pharmthera.2014.12.001

[31] Geenen JJJ, Schellens JHM. Molecular pathways: targeting the protein kinase Wee1 in cancer. Clin Cancer Res. 2017. 10.1158/1078-0432.CCR-17-0520 28442503

[32] Garrett MD, Collins I. Anticancer therapy with checkpoint inhibitors: what, where and when? Trends Pharmacol Sci. 2011;32(5):308–16. doi: 10.1016/j.tips.2011.02.014 21458083 10.1016/j.tips.2011.02.014

[33] Sorensen CS, Hansen LT, Dziegielewski J, Syljuasen RG, Lundin C, Bartek J, et al The cell-cycle checkpoint kinase Chk1 is required for mammalian homologous recombination repair. Nat Cell Biol. 2005;7(2):195–201. doi: 10.1038/ncb1212 15665856 10.1038/ncb1212

[34] Hsieh MC, Thompson T, Wu XC, Styles T, O'Flarity MB, Morris CR, et al The effect of comorbidity on the use of adjuvant chemotherapy and type of regimen for curatively resected stage III colon cancer patients. Cancer Med. 2016;5(5):871–80. PubMed Central PMCID: PMCPMC4864816. doi: 10.1002/cam4.632 26773804 10.1002/cam4.632 PMC4864816

[35] Andre T, de Gramont A, Vernerey D, Chibaudel B, Bonnetain F, Tijeras-Raballand A, et al Adjuvant Fluorouracil, Leucovorin, and Oxaliplatin in Stage II to III Colon Cancer: Updated 10-Year Survival and Outcomes According to BRAF Mutation and Mismatch Repair Status of the MOSAIC Study. J Clin Oncol. 2015;33(35):4176–87. doi: 10.1200/JCO.2015.63.4238 26527776 10.1200/JCO.2015.63.4238

[36] Murakami Y, Uemura K, Hashimoto Y, Kondo N, Nakagawa N, Takahashi S, et al Survival effects of adjuvant gemcitabine plus S-1 chemotherapy on pancreatic carcinoma stratified by preoperative resectability status. J Surg Oncol. 2016;113(4):405–12. doi: 10.1002/jso.24156 26750513 10.1002/jso.24156

[37] Kamisawa T, Wood LD, Itoi T, Takaori K. Pancreatic cancer. Lancet. 2016;388(10039):73–85. doi: 10.1016/S0140-6736(16)00141-0 26830752 10.1016/S0140-6736(16)00141-0

