# Supplementary figures and images for: The G2 checkpoint inhibitor CBP-93872 increases the sensitivity of colorectal and pancreatic cancer cells to chemotherapy
Source: PLoS One. 2017 May 30;12(5):e0178221. doi: 10.1371/journal.pone.0178221 (PMC5448762; doi:10.1371/journal.pone.0178221)

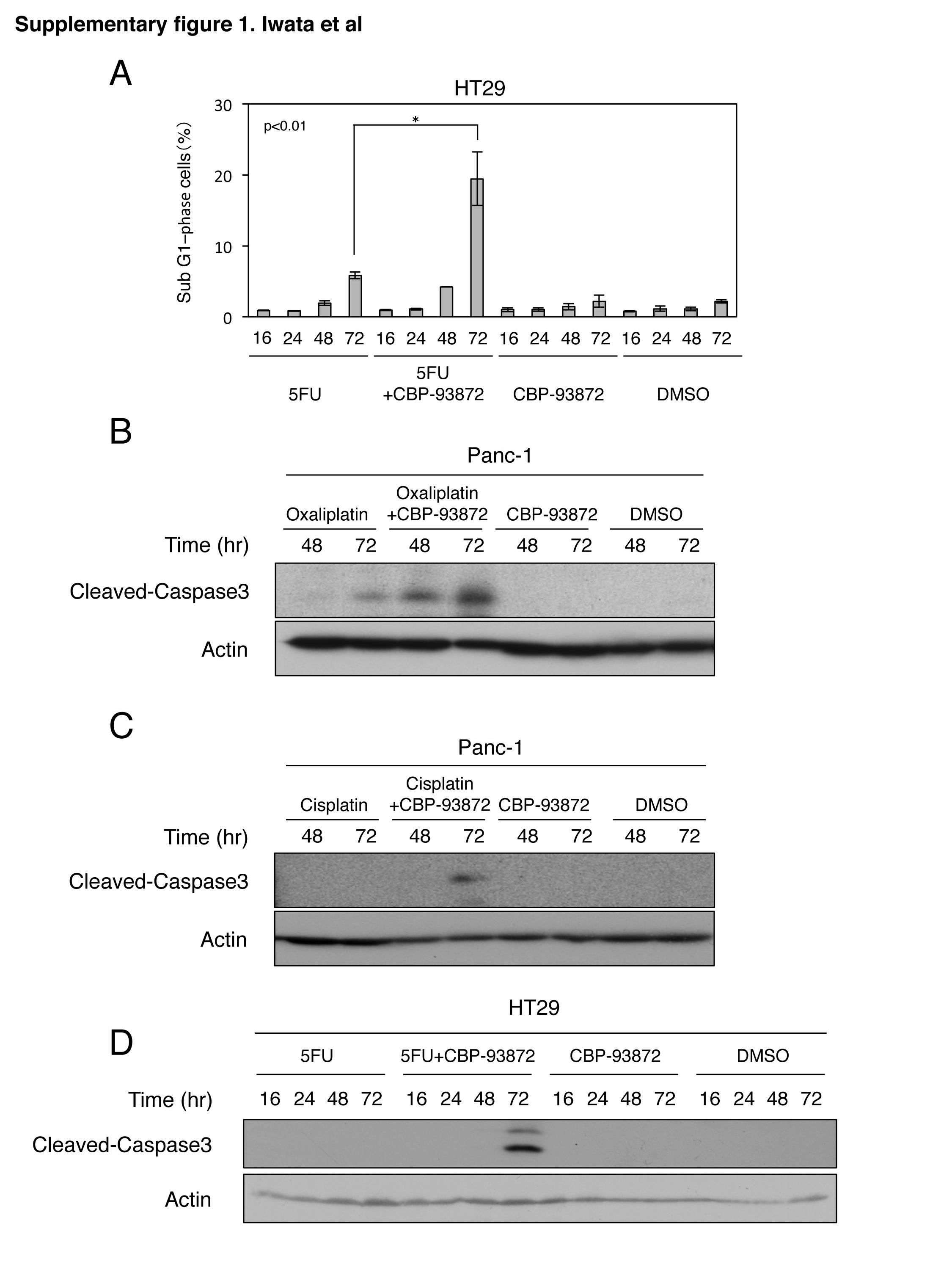

Supplement: S1 Fig — (A) HT29 cells were treated with 5-FU (5 μM), in the presence or absence of CBP-93872 (50 μM). Cells were harvested at the times indicated, fixed and subjected to FACS analysis to determine the proportion of cells in sub-G1 phase. Data are presented as means ± SD (n = 3). Statistical significance was calculated using Student’s t-test (*, p < 0.01). (B, C) Panc-1 cells were treated for the time indicated with oxaliplatin (30 μM) (B), or cisplatin (10 μM) (C), in the presence or absence of CBP-93872 (200 μM). Total cell extracts were analyzed by immunoblotting using the antibodies indicated. (D) HT29 cells were treated and analyzed as in (A). (TIF) [file pone.0178221.s001.tif]

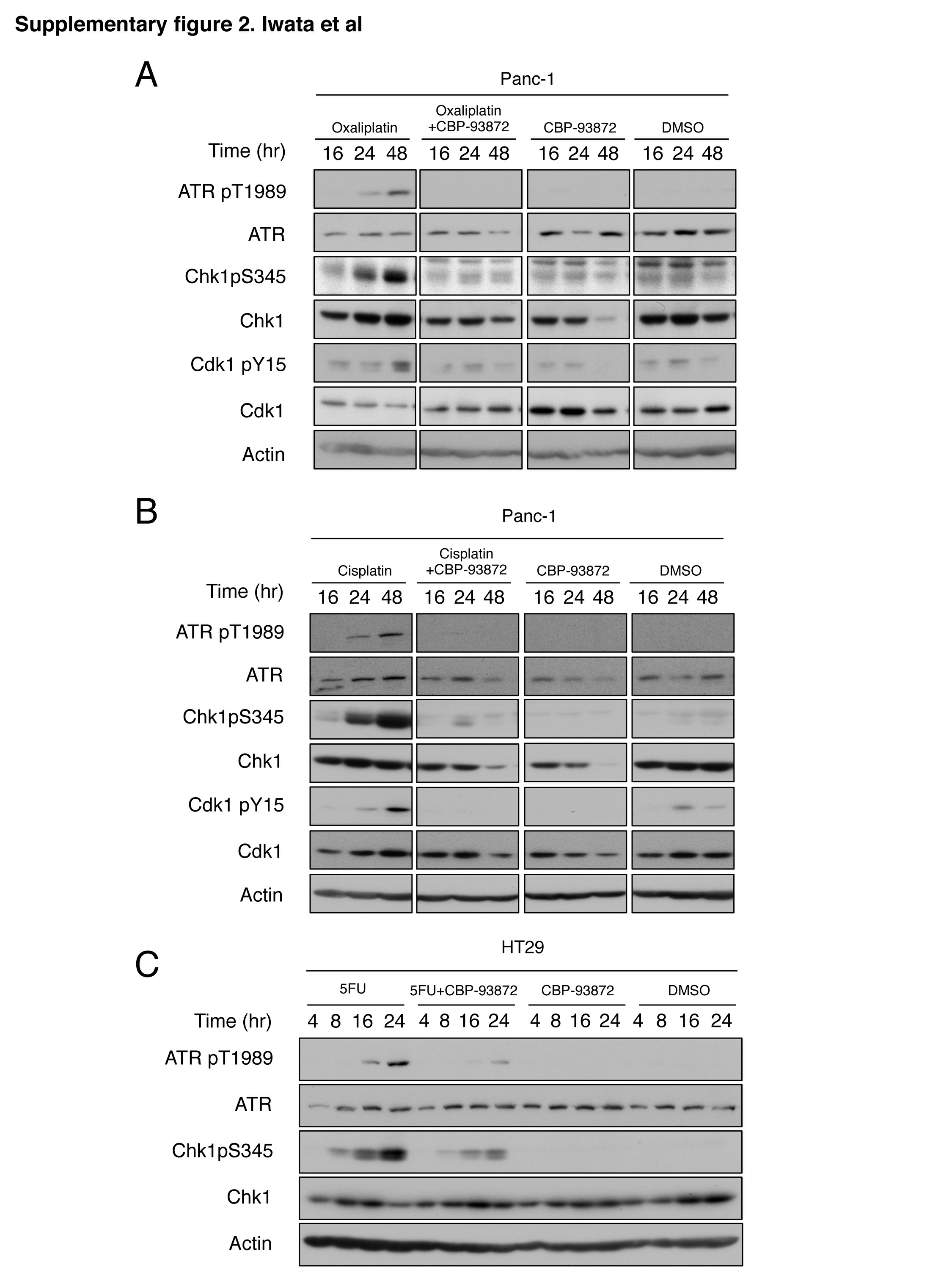

Supplement: S2 Fig — (A) (B) Cells were treated as in S1 Fig, and total cell extracts were subjected to immmunoblotting using indicated antibodies. (C) Experiments were performed as described in S1 Fig, and total cells extracts were subjected to immmunoblotting using indicated antibodies. (TIF) [file pone.0178221.s002.tif]
